# Constituents, pharmacological activities, pharmacokinetic studies, clinical applications, and safety profile on the classical prescription Kaixinsan

**DOI:** 10.3389/fphar.2024.1338024

**Published:** 2024-02-01

**Authors:** Liping Chen, Lin Jiang, Xiaoyu Shi, Jihong Yang, Rong Wang, Wenbin Li

**Affiliations:** ^1^ School of Pharmacy, Gansu University of Chinese Medicine, Lanzhou, Gansu, China; ^2^ Department of Pharmacy, The 940th Hospital of Joint Logistic Support Force of PLA, Lanzhou, China

**Keywords:** Kaixinsan, constituents, pharmacology, pharmacokinetics, clinical applications

## Abstract

Kaixinsan (KXS) is a noteworthy classical prescription, which consists of four Chinese medicinal herbs, namely Polygalae Radix, Ginseng Radix et Rhizoma, Poria, and Acori Tatarinowii Rhizoma. KXS was initially documented in the Chinese ancient book Beiji Qianjin Yaofang written by Sun Simiao of the Tang Dynasty in 652 A.D. As a traditional Chinese medicine (TCM) prescription, it functions to nourish the heart and replenish Qi, calm the heart tranquilize the mind, and excrete dampness. Originally used to treat amnesia, it is now also effective in memory decline and applied to depression. Although there remains an abundance of literature investigating KXS from multiple aspects, few reviews summarize the features and research, which impedes better exploration and exploitation of KXS. This article intends to comprehensively analyze and summarize up-to-date information concerning the chemical constituents, pharmacology, pharmacokinetics, clinical applications, and safety of KXS based on the scientific literature, as well as to examine possible scientific gaps in current research and tackle issues in the next step. The chemical constituents of KXS primarily consist of saponins, xanthones, oligosaccharide esters, triterpenoids, volatile oils, and flavonoids. Of these, saponins are the predominant active ingredients, and increasing evidence has indicated that they exert therapeutic properties against mental disease. Pharmacokinetic research has illustrated that the crucial exposed substances in rat plasma after KXS administration are ginsenoside Re (GRe), ginsenoside Rb1 (GRb1), and polygalaxanthone III (POL). This article provides additional descriptions of the safety. In this review, current issues are highlighted to guide further comprehensive research of KXS and other classical prescriptions.

## 1 Introduction

Due to the distinctive superiority and less adverse effects on the prevention and treatment of sophisticated diseases, Chinese classical prescriptions are attracting more and more attention worldwide (Lu et al., 2021b; Hao et al., 2022). Kaixinsan (KXS) is a noteworthy classical prescription, which was initially described in the Chinese ancient book Beiji Qianjin Yaofang written by Sun Simiao of the Tang Dynasty in 652 A.D. It was originally applied for the treatment of dementia and morbid forgetfulness and is made up of Polygalae Radix (PR), Ginseng Radix et Rhizoma (GR), Poria, and Acori Tatarinowii Rhizoma (ATR) (Zhang et al., 2019). In light of its long history and efficacy, KXS is listed in a catalog of ancient classic prescriptions (the first batch) in China (Chen et al., 2018; Jiang et al., 2021). According to TCM theories, dysfunctions of the spleen cannot nourish the heart, and thus produce dampness to block the heart, which leads to psychiatric diseases with the pivotal symptoms of dizziness, anhedonia, amnesia, and morbid forgetfulness (Pei et al., 2020). KXS has the function of soothing the nerves, replenishing Qi (vital energy), nourishing the heart, and eliminating dampness, thereby improving the dysfunction of the heart and spleen as well as ameliorating diverse forms of mental diseases in clinical settings (Cao et al., 2020; Hu et al., 2021a).

Furthermore, it is known that KXS follows the rule of drug synergism and compatibility in TCM, in which Poria and ATR play adjuvant roles in facilitating the delivery of the principal herb (PR and GR) to the disease site *in vivo*, thus improving the whole prescription to achieve the best effect (Wang et al., 2010; Guo et al., 2019b).

PR, the dried root of the Polygalaceae family member *Polygala tenuifolia* Willd., *Polygala senega* L. or *Polygala sibirica* L. (https://mpns.science.kew.org), is designated as the most essential medication in the prescription (Shan et al., 2023a). Its traditional efficacies include soothing the nerves, improving intelligence, dispelling phlegm, and reducing swelling (Chen et al., 2022). With wide-ranging pharmacological properties that include antidepressant, neuroprotective, hypnotic-sedative, antitumor, antioxidant, anti-inflammatory, antiviral, and antiaging effects, it is broadly used in clinics to treat forgetfulness, depression, insomnia, cough, and palpitation (Jiang et al., 2021; Zhang et al., 2023). Moreover, PR is often combined with Poria, and ATR to by eliminating phlegm and dampness and decreasing gastrointestinal toxicity (Sun et al., 2020).

GR is the dried root of *Panax ginseng* C. A. Meyer of the Araliaceae family (https://mpns.science.kew.org). It is acquainted as the king of herbs because it is widely used as medicinal and functional remedies for miscellaneous diseases in China and other East Asian countries (Li et al., 2021a; Lu et al., 2021a). GR can nourish weakness, replenish energy, invigorate the spleen, benefit the lungs, reinforce vital energy, and consolidate robustness (Liu et al., 2020; Potenza et al., 2023). As the minister medicinal in this prescription, GR is helpful to enter the heart and kidney meridian and enhance the tonifying effect of PR.

**FIGURE 1:**
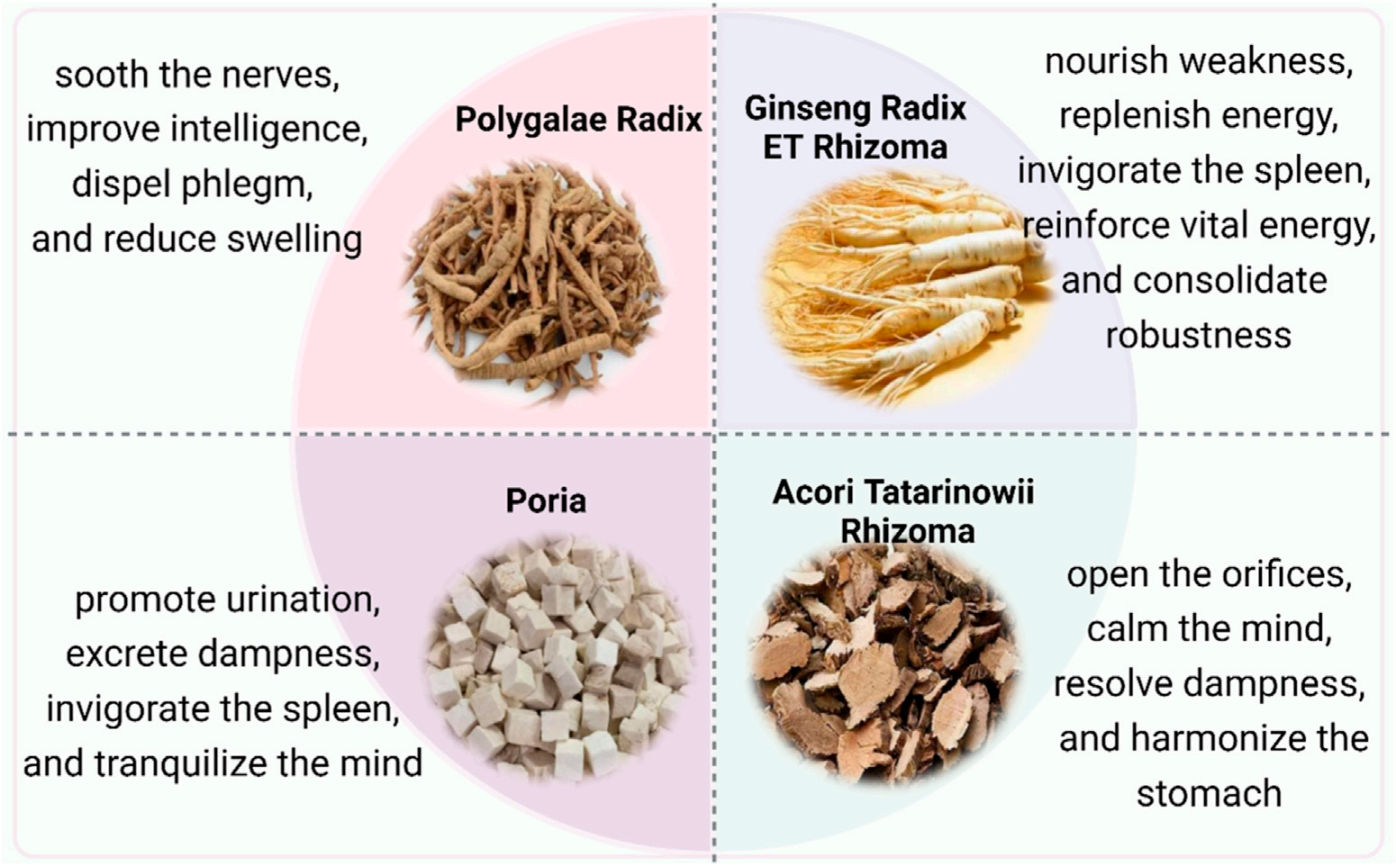
Composition of KXS

Poria and ATR are adjuvant drugs in the prescription. Poria is the dried sclerotium of *Poria cocos* (Schw.) Wolf (Pharmacopoeia Commission of the People’s Republic of China, 2020). It is traditionally used to promote urination, excrete dampness, invigorate the spleen, and tranquilize the mind. ATR is derived from the dried rhizomes of *Acorus tatarinowii* Schott (Pharmacopoeia Commission of the People’s Republic of China, 2020). It can open the orifices (resuscitating), calm the mind, resolve dampness, and harmonize the stomach. Poria cooperates with ATR to strengthen the diuretic effect and sedative activities and thus could promise superior efficacy against amnesia in whole prescription. The Chinese herbal medicine that makes up KXS is illustrated in Figure 1.

Although there are some reports relating to the quality research of KXS, it still failed to form a stable and systematic quality standard system, seriously affecting the therapeutic effects and safety of the prescription (Fu et al., 2019; Bo et al., 2022). Based on the literature to date, the systematic summarization integrating its safety, quality control, and precise mechanisms of action, is still lacking from the point of view of KXS development. Herein, this review discusses current knowledge about the chemical constituents, pharmacological activities, pharmacokinetics, clinical studies, and safety of KXS. It may provide research material and a theoretical foundation for the future study and subsequent development of this classical prescription.

## 2 Chemical constituents

**FIGURE 2:**
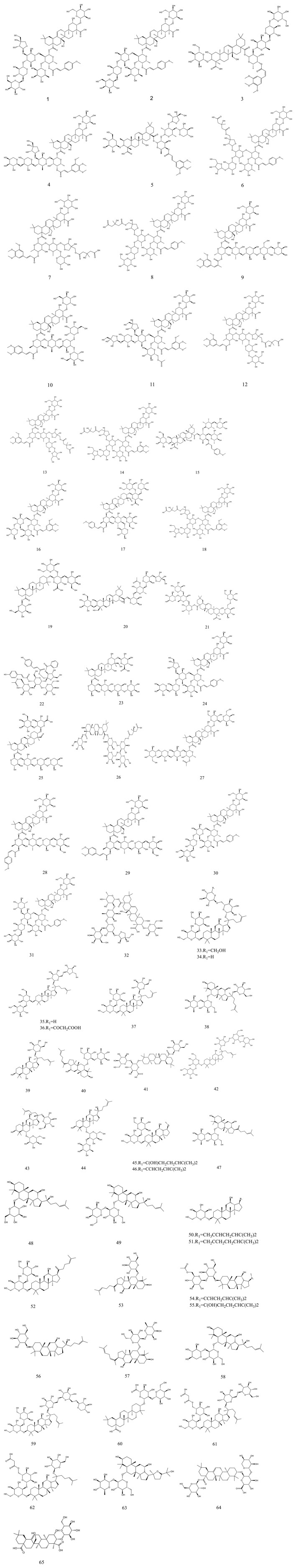
The chemical structures of saponins isolated from KXS.

**FIGURE 3:**
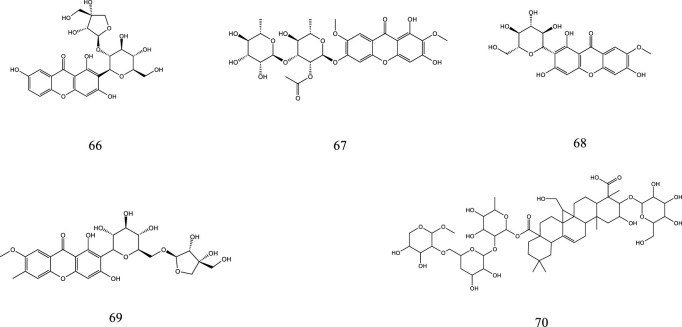
The chemical structures of xanthones isolated from KXS.

**FIGURE 4:**
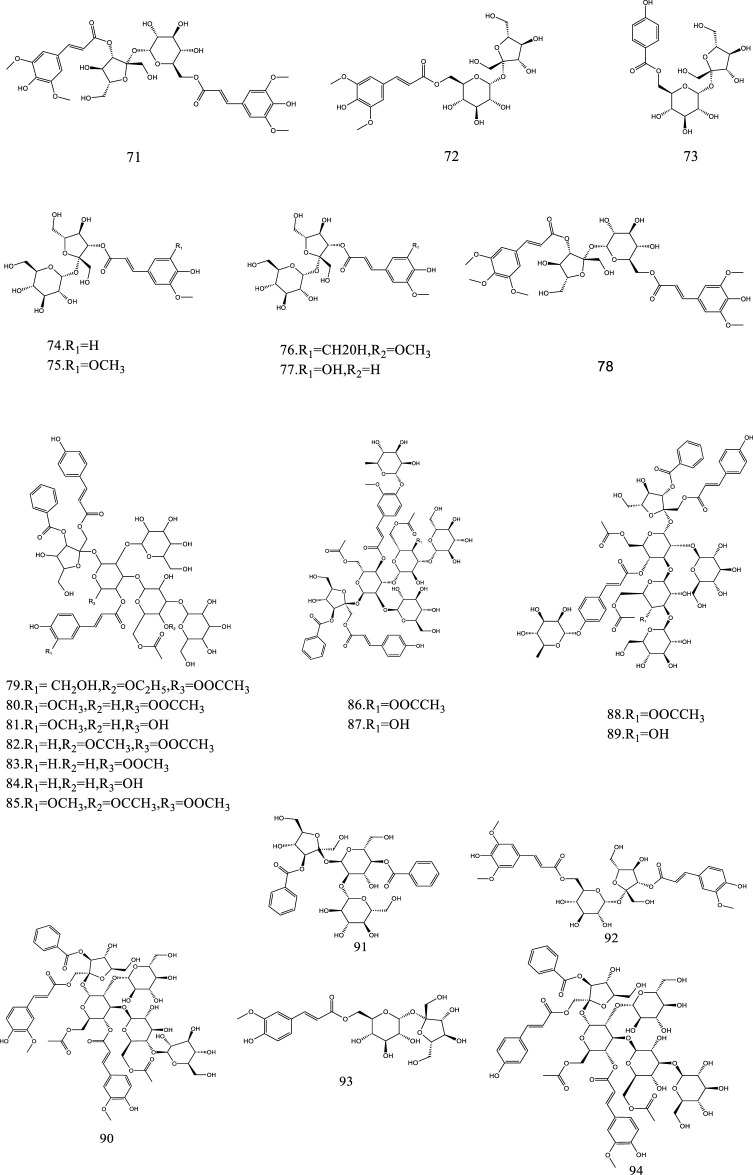
The chemical structures of oligosaccharide esters isolated from KXS.

**FIGURE 5:**
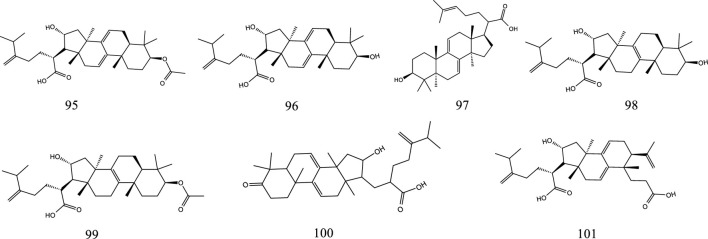
The chemical structures of triterpenoids isolated from KXS.

**FIGURE 6:**
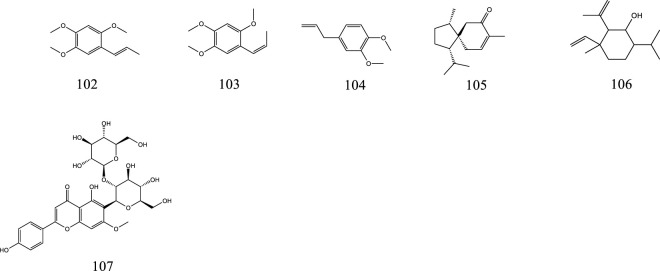
The chemical structures of other compounds isolated from KXS.

So far, 107 chemical compounds have been isolated from KXS. These components are primarily classified into five types, namely, 65 saponins (**1**–**65**), 5 xanthones (**66**–**70**), 24 oligosaccharide esters (**71–94**), 7 triterpenoids (**95–101**), and 6 other types of compounds (**102–107**). Of these, saponins occupy the majority, and more than 60 saponins have been reported. In this article, we summarized all chemical constituents isolated in KXS and showed in Table 1, along with their corresponding structures in Figures 2–6.

**TABLE 1:** Chemical constituents isolated from KXS.

No.	Compounds	Formula	Structure type	Source	References
1	Onjisaponin A	C_80_H_120_O_39_	Saponins	PR	Lv et al. (2016b)
2	Onjisaponin B	C_75_H_112_O_35_	Saponins	PR	Lv et al. (2016b)
3	Onjisaponin E	C_71_H_106_O_33_	Saponins	PR	Lv et al. (2016b)
4	Onjisaponin F	C_75_H_112_O_36_	Saponins	PR	Lv et al. (2016b)
5	Onjisaponin G	C_70_H_104_O_32_	Saponins	PR	Lv et al. (2016b)
6	Onjisaponin J	C_85_H_126_O_42_	Saponins	PR	Lv et al. (2016b)
7	Onjisaponin K	C_76_H_112_O_36_	Saponins	PR	Lv et al. (2016b)
8	Onjisaponin L	C_86_H_128_O_43_	Saponins	PR	Lv et al. (2016b)
9	Onjisaponin O	C_77_H_116_O_37_	Saponins	PR	Lv et al. (2016b)
10	Onjisaponin R	C_76_H_114_O_37_	Saponins	PR	Lv et al. (2016b)
11	Onjisaponin T	C_83_H_124_O_42_	Saponins	PR	Lv et al. (2016b)
12	Onjisaponin V	C_82_H_122_O_41_	Saponins	PR	Lv et al. (2016b)
13	Onjisaponin W	C_81_H_120_O_40_	Saponins	PR	Lv et al. (2016b)
14	Onjisaponin X	C_87_H_130_O_45_	Saponins	PR	Lv et al. (2016b)
15	Onjisaponin Y	C_69_H_102_O_30_	Saponins	PR	Lv et al. (2016b)
16	Onjisaponin Z	C_71_H_106_O_32_	Saponins	PR	Lv et al. (2016b)
17	Onjisaponin Ng	C_80_H_118_O_38_	Saponins	PR	Lv et al. (2016b)
18	Onjisaponin Sg	C_87_H_130_O_44_	Saponins	PR	Liu and Ye (2012)
19	Polygalasaponin ⅩⅣ	C_54_H_86_O_24_	Saponins	PR	Reeju et al., (2017)
	ⅩⅩⅣ				
20	polygalasaponin ⅩⅧ	C_59_H_94_O_28_	Saponins	PR	Reeju et al., (2017)
21	Polygalasaponin ⅩⅨ	C_64_H_102_O_32_	Saponins	PR	Reeju et al., (2017)
22	Polygalasaponin ⅩⅩⅠ	C_51_H_60_O_27_	Saponins	PR	Reeju et al., (2017)
	ⅩⅩⅠ				
23	Polygalasaponin ⅩⅩⅡ	C_58_H_92_O_28_	Saponins	PR	Reeju et al., (2017)
24	Polygalasaponin XXXII	C_79_H_118_O_38_	Saponins	PR	Liu and Ye (2012)
25	Polygalasaponin ⅩⅩⅩⅤ	C_63_H_98_O_31_	Saponins	PR	Reeju et al., (2017)
26	Arilloside E	C_66_H_104_O_34_	Saponins	PR	Reeju et al., (2017)
27	Polygalasaponin ⅩⅩⅩⅫ	C_67_H_106_O_35_	Saponins	PR	Reeju et al., (2017)
28	Senegasaponin B	C_69_H_102_O_31_	Saponins	PR	Reeju et al., (2017)
29	Senegin Ⅱ	C_70_H_104_O_32_	Saponins	PR	Reeju et al., (2017)
30	Senegin III	C_75_H_112_O_35_	Saponins	PR	Lv et al. (2016b)
31	Senegin IV	C_81_H_122_O_39_	Saponins	PR	Liu and Ye (2012)
32	Arillatanoside C	C_64_H_102_O_33_	Saponins	PR	Lv et al. (2016b)
33	Ginsenoside Rb1	C_54_H_92_O_23_	Saponins	GR	Lv et al. (2016b)
34	Ginsenoside Rb2	C_53_H_90_O_22_	Saponins	GR	Lv et al. (2016b)
35	Ginsenoside Rc	C_53_H_90_O_22_	Saponins	GR	Lv et al. (2016b)
36	Ginsenoside mRc	C_56_H_92_O_25_	Saponins	GR	Lv et al. (2016b)
37	Ginsenoside Rd	C_48_H_82_O_18_	Saponins	GR	Lv et al. (2016b)
38	Ginsenoside Re	C_48_H_82_O_18_	Saponins	GR	Lv et al. (2016b)
39	Ginsenoside F1	C_36_H_62_O_9_	Saponins	GR	Lv et al. (2016b)
40	Ginsenoside F4	C_42_H_70_O_12_	Saponins	GR	Lv et al. (2016b)
41	Ginsenoside Ro	C_48_H_76_O_19_	Saponins	GR	Lv et al. (2016b)
42	Ginsenoside Rg1	C_42_H_72_O_14_	Saponins	GR	Lv et al. (2016b)
43	Ginsenoside Rg2	C_42_H_72_O_13_	Saponins	GR	Lv et al. (2016b)
44	20SGinsenoside Rg3	C_42_H_72_O_13_	Saponins	GR	Lv et al. (2016b)
45	20RGinsenoside Rg3	C_42_H_72_O_13_	Saponins	GR	Lv et al. (2016b)
46	Ginsenoside Rg5	C_42_H_70_O_12_	Saponins	GR	Lv et al. (2016b)
47	Ginsenoside Rg6	C_42_H_70_O_12_	Saponins	GR	Lv et al. (2016b)
48	Ginsenoside Rh1	C_36_H_62_O_9_	Saponins	GR	Lv et al. (2016b)
49	Ginsenoside Rf	C_42_H_72_O_14_	Saponins	GR	Lv et al. (2016b)
50	Ginsenoside Rh3	C_36_H_60_O_7_	Saponins	GR	Lv et al. (2016b)
51	Ginsenoside Rk1	C_42_H_70_O_12_	Saponins	GR	Lv et al. (2016b)
52	Ginsenoside Rk2	C_36_H_60_O_7_	Saponins	GR	Lv et al. (2016b)
53	Ginsenoside Rk3	C_36_H_60_O_8_	Saponins	GR	Lv et al. (2016b)
54	Ginsenoside Rs3	C_44_H_74_O_14_	Saponins	GR	Lv et al. (2016b)
55	Ginsenoside Rs4	C_44_H_72_O_13_	Saponins	GR	Lv et al. (2016b)
56	Ginsenoside Rh2	C_36_H_62_O_8_	Saponins	GR	Lv et al. (2016b)
57	Ginsenoside F4/Rg4	C_42_H_70_O_12_	Saponins	GR	Lv et al. (2016b)
58	Notoginsenoside R_2_	C_41_H_70_O_13_	Saponins	GR	Liu et al. (2005)
59	Notoginsenoside R_4_	C_59_H_100_O_27_	Saponins	GR	Liu et al. (2005)
60	Zingibroside R_1_	C_42_H_66_O_14_	Saponins	GR	Lv et al. (2016b)
61	Malonyl-ginsenoside Rb1	C_57_H_94_O_26_	Saponins	GR	Lv et al. (2016b)
62	Malonyl-ginsenoside Rd	C_51_H_84_O_21_	Saponins	GR	Lv et al. (2016b)
63	Pseudoginsenoside F11	C_42_H_72_O_14_	Saponins	GR	Lv et al. (2016b)
64	Pseudoginsenoside RT1	C_47_H_74_O_18_	Saponins	GR	Lv et al. (2016b)
65	Tenuifolin	C_36_H_56_O_12_	Saponins	PR	Lv et al. (2016b)
66	Sibiricaxanthone B	C_24_H_26_O_14_	Xanthones	PR	Lv et al. (2016b)
67	Sibiricaxanthone F	C_29_H_34_O_16_	Xanthones	PR	Lv et al. (2016b)
68	7-O-Methylmangiferin	C_20_H_20_O_11_	Xanthones	PR	Lv et al. (2016b)
69	Polygalaxanthone III	C_25_H_28_O_15_	Xanthones	PR	Wang et al. (2021b)
70	Polygalaxanthone XXVIII	C_53_H_84_O_24_	Xanthones	PR	Lv et al. (2016b)
71	3,6′-disinapoyl sucrose	C_34_H_42_O_19_	Oligosaccharide esters	PR	Lv et al. (2016a); Lin et al. (2021)
72	Sibiricose A1	C_23_H_32_O_15_	Oligosaccharide esters	PR	Lv et al. (2016b)
73	Sibiricose A3	C_19_H_26_O_13_	Oligosaccharide esters	PR	Wang et al. (2017b)
74	Sibiricose A5	C_22_H_30_O_14_	Oligosaccharide esters	PR	Lv et al. (2016a)
75	Sibiricose A6	C_23_H_32_O_15_	Oligosaccharide esters	PR	Lv et al. (2016a)
76	Tenuifoliside A	C_31_H_38_O_17_	Oligosaccharide esters	PR	Lv et al. (2016b); Lin et al. (2021)
77	Tenuifoliside B	C_30_H_36_O_17_	Oligosaccharide esters	PR	Lv et al. (2016b)
78	Tenuifoliside C	C_35_H_44_O_19_	Oligosaccharide esters	PR	Lv et al. (2016b)
79	Tenuifoliose A	C_62_H_76_O_35_	Oligosaccharide esters	PR	Lv et al. (2016b)
80	Tenuifoliose B/D	C_60_H_74_O_34_	Oligosaccharide esters	PR	Lv et al. (2016b)
81	Tenuifoliose E/C	C_58_H_72_O_33_	Oligosaccharide esters	PR	Lv et al. (2016b)
82	Tenuifoliose H	C_61_H_74_O_34_	Oligosaccharide esters	PR	Lv et al. (2016b)
83	Tenuifoliose I/J	C_59_H_72_O_33_	Oligosaccharide esters	PR	Lv et al. (2016b)
84	Tenuifoliose K	C_57_H_70_O_32_	Oligosaccharide esters	PR	Lv et al. (2016b)
85	Tenuifoliose N	C_63_H_78_O_36_	Oligosaccharide esters	PR	Wang et al. (2017a)
86	Tenuifoliose F	C_68_H_86_O_39_	Oligosaccharide esters	PR	Lv et al. (2016b)
87	Tenuifoliose G	C_66_H_84_O_38_	Oligosaccharide esters	PR	Lv et al. (2016b)
88	Tenuifoliose L	C_67_H_84_O_38_	Oligosaccharide esters	PR	Lv et al. (2016b)
89	Tenuifoliose M	C_65_H_82_O_37_	Oligosaccharide esters	PR	Lv et al. (2016b)
90	Tenuifoliose O	C_61_H_76_O_35_	Oligosaccharide esters	PR	Lv et al. (2016b)
91	Telephiose C	C_32_H_40_O_18_	Oligosaccharide esters	PR	Lv et al. (2016b)
92	Arillanin A	C_33_H_40_O_18_	Oligosaccharide esters	PR	Wang et al. (2017b)
93	Arillanin C	C_22_H_30_O_14_	Oligosaccharide esters	PR	Wang et al. (2017a)
94	Reiniose J	C_60_H_74_O_34_	Oligosaccharide esters	PR	Wang et al. (2017b)
95	Dehydropachymic acid	C_33_H_50_O_5_	Triterpenoids	Poria	Lv et al. (2016b)
96	Dehydrotumulosic acid	C_31_H_48_O_4_	Triterpenoids	Poria	Wang et al. (2021b)
97	Dehydrotrametenolic acid	C_30_H_46_O_3_	Triterpenoids	Poria	Wang et al. (2021b)
98	Tumulosic acid	C_31_H_50_O_4_	Triterpenoids	Poria	Lv et al. (2016a)
99	Pachymic acid	C_33_H_52_O_5_	Triterpenoids	Poria	Wang et al. (2021b)
100	Polyporenic acid C	C_31_H_46_O_4_	Triterpenoids	Poria	Lin et al. (2021)
101	Poricoic acid A	C_31_H_46_O_5_	Triterpenoids	Poria	Lin et al. (2021)
102	α-asarone	C_12_H_16_O_3_	Other compounds	ATR	Wang et al. (2021a)
103	β-asarone	C_12_H_16_O_3_	Other compounds	ATR	Wang et al. (2021a)
104	Methyleugenol	C_11_H_14_O_2_	Other compounds	ATR	Lin et al. (2021)
105	Acorenone B	C_15_H_24_O	Other compounds	ATR	Lin et al. (2021)
106	Shyobunol	C_15_H_26_O	Other compounds	ATR	Lin et al. (2021)
107	Spinosin	C_28_H_32_O_15_	Other compounds	ATR	Lin et al. (2021)

### 2.1 Saponins

Saponins are a diverse category of natural compounds that contain one or more sugar chains and a triterpene or steroid aglycone in the basic parent nucleus. This particular category of natural components is a broad-scale distribution in Chinese herbs. Saponins are the predominant active ingredient of KXS, and increasing evidence has indicated that they exert therapeutic properties against memory and cognitive impairment, depression, and neurodegenerative diseases (Lu et al., 2022; Qiu et al., 2022). To date, a total of 65 saponins have been reported in KXS, which mostly consist of polygala saponins and ginsenosides. Structurally, ginsenosides are further classified into four major categories based on the parent ring structure, including the protopanaxadiol type (**33**–**37**, **44**–**46**, **50**–**52**, **54**–**57**), the protopanaxatriol type (**38**–**40**, **42**, **43**, **47**–**49**, **53**), the oleanane group (**41**) and pseudoginsenosides of the ocotillol type (**63**, **64**).

### 2.2 Xanthones

The name xanthones designates a class of secondary metabolites normally found in a few higher plant families. The polygalaceae family is an important source of natural xanthones which have the basic mother nucleus of 9*H*-xanthen-9-one or xanthone. Currently, 5 xanthones from KXS have been identified, including sibiricaxanthone B (66), sibiricaxanthone F (67), 7-O-methylmangiferin (68), polygalaxanthone III (69), and polygalaxanthone XXVIII (70).

### 2.3 Oligosaccharide esters

Oligosaccharide esters are a sizable class of natural metabolites with sucrose and trisaccharide esters as a character in their chemical structures and have a variety of physiological activities. At present, a total of 24 oligosaccharide esters have been reported in KXS.

### 2.4 Triterpenoids

There are currently 7 reported triterpenoids that have been identified from KXS. Poria is rich sources of triterpenoids.

### 2.5 Other compounds

In addition to the above ingredients, KXS also contains volatile oils (**102**–**106**) and flavonoids (**107**).

## 3 Pharmacological activities

Traditionally, KXS has the effect of replenishing Qi, nourishing the heart, and calming the mind. Therefore, it has been used clinically to treat a range of nervous system conditions. Modern studies on KXS have been carried out to demonstrate pharmacological effects. The pharmacological effects are shown in Table 2.

**TABLE 2:** Pharmacological effects of KXS.

Pharmacological activity	Model	Effective dose	Route	Positive control	Effects	Mechanisms	Application	References
Anti-AD effects	Male SD rats AD model; PC12 cells	10 g/kg	p.o.	Huperzine A 30 ug/kg	Regulate the levels of central neurotransmitters, reducing the neuroinflammation and ROS-induced neuronal apoptosis via PI3K/Akt/GSK-3β pathways.	Ach ↑	*In vivo* and *in vitro*	Guo et al. (2019c)
						GABA ↑		
						DA ↑		
						5-HT ↑		
						5-HIAA ↑		
						p-PI3K ↑		
						p-Akt ↑		
						p-GSK-3β ↑		
						p-Tau↓		
						ROS ↓		
						IL-1β ↓		
						TNF-α ↓		
						AA ↓		
						Bax ↓		
						Bcl-2 ↑		
						Caspase-3 ↓		
	Kun-Ming mice AD model	1.4, 2.8 g/kg	p.o.	Donepezil 3 mg/kg	Inhibit the inflammation of microglia, alleviate cholinergic system dysfunction.	IL-1β ↓	*In vivo*	Luo et al. (2020)
						TNF-α ↓		
						IL-6 ↓		
						CHRNB2 ↑		
	Male SAMP8 mice	5.4 g/kg	p.o.		Attenuate Tau hyperphosphorylation and neuroinflammation by inhibiting the TLR4/MyD88/NF-κB signaling pathway, therefore suppressing neuronal apoptosis and improving the cognitive function.	Tau phosphorylation ↓	*In vivo*	Jiao et al. (2022)
						p-GSK3β (Tyr216)/GSK3β ↓		
						p-GSK3β (Ser9)/GSK3β ↑		
						p-AKT (S473)/AKT ↑		
						TLR4 ↓		
						MyD88 ↓		
						NF-κB ↓		
						Bax ↓		
						Bcl-2 ↑		
						Caspase 1 ↓		
						Caspase 3 ↓		
						MMP ↑		
						ATP ↑		
						EC ↑		
						Glutamate ↓		
						p-NMDAR1 ↓		
						Ptch1 ↑		
						Smo ↑		
						Gli1 ↑		
The effects on memory and cognitive functions	Male Kunming mice model of scopolamine-induced cognitive dysfunction	0.7, 1.4, 2.8 g/kg	p.o.	Donepezil 3 mg/kg	Improve cognitive function through reducing apoptosis and oxidative stress, and regulating synapse-associated protein and cholinergic neurotransmitters.	Bcl-2 ↑	*In vivo*	Xu et al. (2019)
						Bax ↓		
						PSD 95 ↑		
						SYN ↑		
						BDNF ↑		
						ChAT ↑		
						ACh ↑		
						AChE ↓		
						SOD ↑		
						GSH-Px ↑		
						ROS ↓		
						MDA ↓		
	Male SD rat model of PSD-induced cognitive deficit	125, 250, 500 mg/kg	p.o.	Modafinil 30 mg/kg	Improve cognitive function by modulation of neurotransmitter levels and the expression of some genes in the brain that contribute to memory functions.	GLU ↓	*In vivo*	Hu et al. (2013)
						GABA ↓		
						BDNF ↑		
						CREB ↑		
						p-CREB ↑		
	Male SD rat model of simulated weightless-ness	0.3, 0.6 g/kg	p.o.	Huperzine A, 0.1 mg/kg	Improve memory deficiency by inhibiting oxidative stress damage	ROS ↓	*In vivo*	Qiong et al. (2016)
						8-OHdG ↓		
						3-NT ↓		
	Female BALB/c mice model of chemotherapy-induced cognitive impairment	1 g/kg	p.o.	Doxorubicin, 5 mg/kg	Regulate inflammatory responses and reduce oxidative stress and neural degeneration.	IL-6 ↓	*In vivo*	Lyu et al. (2021)
						IL-12p70 ↓		
						IL-1β ↓		
						TFN-α ↓		
						IL-4 ↑		
						IL-10 ↑		
						MDA ↓		
						SOD ↑		
						GPx ↑		
						CAT ↑		
						GSH ↑		
Anti-depression effects	Male Wistar rats model of CUMS-induced depression; LPS- induced rat astrocytes	3, 5, 10 g/kg	p.o.	Fluoxetine, 10 mg/kg	Ameliorate depressive behaviors and inhibited the NLRP3 inflammasome-mediated inflammation in vivo and in vitro by inducing autophagy.	IL-6 ↓	*In vivo* and *in vitro*	Yu et al. (2021)
						IL-1β ↓		
						TNF-α ↓		
						NLRP3 ↓		
						ASC ↓		
						Caspase-1 ↓		
						MDA ↓		
						SOD ↑		
						GSH ↑		
						LC3-II ↑		
						P62 ↓		
						Beclin1 ↑		
						ROS ↓		
	Male ICR mice model of CUMS-induced depression	3, 10 g/kg	p.o.	Fluoxetine 4 mg/kg	Modulate gut micro-environment modification, suppress neuronal inflammation in the brain and inhibition of HPA axis activation.	Allobaculum ↑	*In vivo*	Cao et al. (2020)
						Bifidobacterium ↑		
						Turicibacter ↑		
						Coprococcus ↓		
						Mucispirillum ↓		
						Odoribacter ↓		
						Oscillospira ↓		
						IL-6 ↓		
						IL-1β ↓		
						TNF-α ↓		
						ZO-1 ↑		
						Occludin ↑		
						Claudin-5 ↑		
						ACTH ↓		
						Corticosterone ↓		
	Male rat model of depression with MI	370 mg/kg	p.o.		Possesse both antidepressive effects and cardioprotective functions	CK-MB ↓	*In vivo*	Hu et al. (2020)
						LDH ↓		
						SOD ↑		
						GSH-PX ↑		
						CAT ↑		
						MDA ↓		
	Male KM mice model of depression	175, 350, 700, 1,400 mg/kg	p.o.	Fluoxetine 28 mg/kg	Regulate the central monoaminergic neurotransmitter system	5-HT ↑	*In vivo*	Zhou et al. (2012)
						DA ↑		
						NE ↑		
	Male ICR mice model of CUMS-induced depression	1.5 g/kg	p.o.	Fluoxetine 4 mg/kg	Regulate neurotrophin-Trk signaling pathway	NGF ↑	*In vivo*	Zhu et al. (2017)
						BDNF ↑		
						Trk receptors ↑		
	Male wistar rat model of CUMS-induced depression; primary cultures of rat hippocampal neuron	1.5, 5 g/kg	p.o.	Fluoxetine 3.6 mg/kg	Beneficial for synaptogenesis by inducing synaptic protein expressions via cAMP-dependent signaling pathway.	Synapsin II ↑	*In vivo* and *in vitro*	Zhu et al. (2016)
						Synaptotagmin ↑		
						PSD95 ↑		
						Bt2-cAMP ↑		
	Male ICR mice model of CUMS-induced depression; Mice microglial cell lines (BV2)	3, 10 g/kg	p.o.	Fluoxetine 7.2 mg/kg	Decrease pro-inflammatory cytokine expression in microglia via inhibiting TLR4/IKK/NF-КB pathways.	IL-1β ↓	*In vivo* and *in vitro*	Qu et al. (2021)
						IL−2 ↓		
						TNF-α ↓		
						TLR4 ↓		
						p-NF-КB/NF-КB↓		
						p-IKK/IKK ↓		
	Mouse astrocytes	1–10 ug/mL			Increase expressions of synthesis kinase tPA in neurotrophic factor metabolic pathway	NGF ↑	*in vitro*	Cao et al. (2018)
						BDNF ↑		
						Bt2-cAMP ↑		
						p-CREB ↑		
Anti-fatigue effects	Male SD rats model of fatigue induced by forced wheel running	125, 250, 500 mg/kg	p.o.	Modafinil, 13 mg/kg	Increase exhaustive running time in the treadmill running test	Hepatic/muscle glycogen ↑	*In vivo*	Cao et al. (2012)
						Testosterone ↑		
						LDH ↓		
						SUN ↓		
						BLA ↓		
						β-endorphin ↓		
						SOD ↑		
						MDA ↓		

### 3.1 Anti-Alzheimer’s disease effects

**FIGURE 7:**
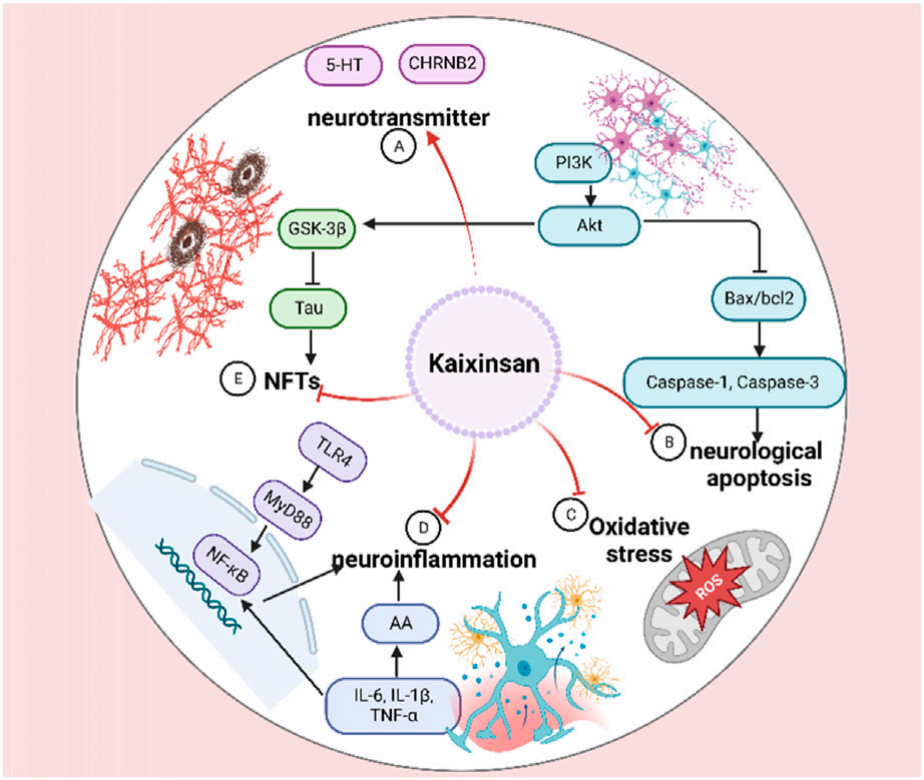
Anti-Alzheimer’s Disease mechanism of Kaixinsan

Alzheimer’s disease (AD) is the most common neurodegenerative disease, manifesting progressive cognitive impairment, amnesia, and mental behavioral changes (Conti Filho et al., 2023). The curative effects of KXS on amnesia have been verified throughout millennia. It is widely accepted that the gradual enrichment of Amyloid-β (Aβ) peptide as well as abnormal or hyperphosphorylated Tau are strongly correlated with AD pathogenesis (Khayat et al., 2021; Wegmann et al., 2021; Zhang et al., 2021). As shown in Figure 7, KXS has been proven to possess excellent anti-AD effects by inhibiting neuroinflammation, neurological apoptosis, and oxidative stress, and increasing the supply of neurotransmitters.

AD was induced in male SD rats through an intraperitoneal injection of D-gal (50 mg/kg/d) and a stereotaxic injection of 4 μg Aβ_25-35_ (1 μg/μL) for 6 weeks. The rats were orally administered KXS water extracts (10 g/kg/d) or huperzine A (30 μg/kg) from the third week. The spatial learning and memory were measured with the escape latency, platform crossings, and swimming distance to find the removal platform in the target quadrant of the Morris water maze. Neuronal density in hippocampal CA1 regions was performed using immunohistochemistry staining. According to the results of all the detection indexes, the water extract observably decreased the escape latency and swimming distance, increased platform crossings, increased fluorescence intensity of neuronal nuclei, ameliorated central neurotransmitter loss, reduced Tau hyperphosphorylation via regulating the expression of PI3K/Akt. Moreover, the extract dramatically decreased the concentrations of inflammatory factors and ROS-induced neuronal apoptosis via the PI3K/Akt/GSK-3β signaling pathway in the rat hippocampus. *In vitro*, the extracts potently inhibited the percentage of apoptotic cells and the expressions of Bax/bcl-2 and cleaved-caspase-3, meliorated Aβ_25-35_-induced ROS accumulation and LDH release, and reduced the expression of Tau signaling pathway in PC12 cells (at the dose of 1 mg/mL). Based on the results of the *in vivo* and *in vitro* experiments, KXS extracts indicated its potentially beneficial role in ameliorating AD progression (Guo et al., 2019c). An *in vivo* study on AD rats after treatment with KXS indicated that 23 constituents were found in AD rats’ plasma, including 1 from PR, 8 from GR, 4 from Poria, 6 from ATR, and 4 that were only discovered in KXS. meanwhile, 10 components were detected in AD rats’ brains, including 4 from GR and 6 that were only existed in the whole prescription. Furthermore, it was found that the peak areas of some constituents in the KXS group were bigger than those in the single herbs group *via* semiquantitative analysis. KXS might mediate oxidative stress, neuroinflammation, and apoptosis both *in vivo* and *in vitro*, especially when the four single herbs were adopted in combination. These results demonstrated the synergistic function and the whole prescription compatibility rationality of KXS to a certain degree (Guo et al., 2019a).

Meta-analysis of animal experiments is believed to provide a high level of research evidence (Chou et al., 2020). Network pharmacology is helpful in elucidating the mechanisms of the TCM formula in the treatment of complex diseases in a comprehensive and systematic manner (Zhou et al., 2022a). To further elucidate the active ingredients and the mechanisms of KXS, Yi et al. (2020) employed meta-analysis, network pharmacology analyses, and molecular docking to investigate the efficacy and potential underlying action mechanisms of KXS against AD from a holistic prospect. The result indicated that aposcopolamine, stigmasterol, and inermin exhibited good affinity for several key genes associated with memory and cognition, such as muscarinic acetylcholine receptors, adrenergic receptors, and acetylcholinesterase (AChE). Scopolamine-induced amnesia is an excellent model of behavioural-cum-cognitive deficits to study dementing-related illnesses such as AD (Tang, 2019). In scopolamine (3 mg/kg/d i. p.)-induced cognitive dysfunction mice, Luo et al. (2020) validated the anti-inflammatory effects of the ethanol extracts of KXS (at doses of 1.4 and 2.8 g/kg) by decreasing proinflammatory cytokine levels such as tumor necrosis factor (TNF)-α, interleukin (IL)-6 and IL-1β both in the hippocampus and cortex, which were consistent with the network pharmacology analyses. More importantly, it was found that cholinergic system dysfunction improvement of KXS was significantly correlated with regulation of the nicotinic acetylcholine receptor CHRNB2. Senescence accelerated mouse prone 8 (SAMP8) was reported to develop typical AD pathologies, such as an abnormal expression of anti-aging factors, inflammation, oxidative stress, tau hyperphosphorylation, amyloid-(A) deposits, abnormal autophagy activity, and endoplasmic reticulum stress (Andrea et al., 2022). Therefore, SAMP8 is widely used and considered as an ideal model for AD research, displaying age-related cognitive decline and memory loss (Xie et al., 2020). In a subsequent study, the potential molecular mechanisms of KXS on AD which was predicted by the network pharmacology approach were experimentally validated in SAMP8. The result indicated that the water extract (at doses of 5.4 g/kg) significantly upregulated AKT phosphorylation, inhibited the activation of GSK3β and CDK5, and suppressed the TLR4/MyD88/NF-КB pathway to suppress neuronal apoptosis and attenuate cognitive impairment, thus alleviating the progression of AD (Jiao et al., 2022). Although dozens of *in vivo* and *in vitro* experiments of KXS and its components have supported the anti-AD potentials, explicit pharmacokinetic clinical trials should be executed to further explore the principles of formulating prescriptions and elucidate the correlation between bioactive ingredients and pharmacological function.

### 3.2 Anti-depression effects

Depression, a common but devastating psychiatric illness characterized by anhedonia, despair, anxiety, cognitive changes, and sleep disturbance, has become a pressing public health problem that leads to a heavy socioeconomic burden (Cladder-Micus et al., 2018). More than 264 million people worldwide are suffering from this debilitating illness (Monroe and Harkness, 2022). Unfortunately, depression is an extremely complex disease caused by multiple factors, and the actual etiology of this disease is still unclarified (McCarron et al., 2021). Nowadays, substantial basic research is ongoing to shed light on depression. Many pathological hypotheses including brain-derived neurotrophic factor (BDNF), neurotransmitter imbalance, over-stimulation of inflammatory, hypothalamic-pituitary-adrenal (HPA) axis disturbances, and endogenous metabolites, have been put forward (Fox and Lobo, 2019).

**FIGURE 8:**
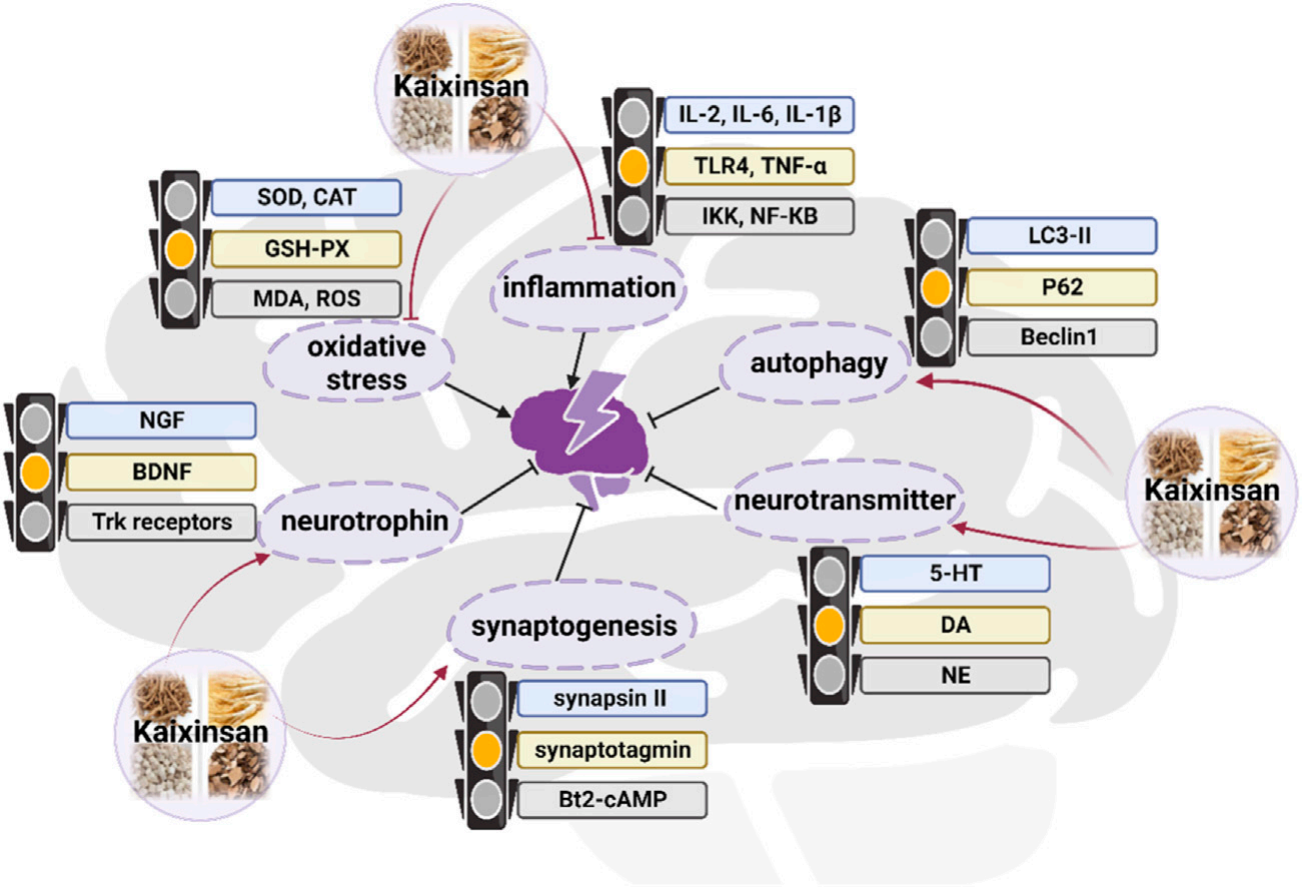
Anti-depression mechanism of Kaixinsan

In terms of TCM theory, depression is believed to be caused by the stagnant of Qi (vital energy), moisture, phlegm, or heat, which ultimately leads to brain dysfunction (Li et al., 2023). KXS has the function of tonifying Qi, nourishing the heart, eliminating dampness, and resolving phlegm, thereby for the treatment of depression function. An increasing number of studies have suggested that KXS was used to effectively treat depression by regulating autophagy, neurotrophic factors, neurotransmitters, synaptogenesis, and inflammation *in vivo* and *in vitro* experiments (Figure 8) (Zhou et al., 2012; Zhu et al., 2012; Zhu et al., 2013; Dong et al., 2017; Fu et al., 2019). Emerging experimental investigations have suggested that chronic stress which causes many impairments in mood, memory, and cognition, has been considered to play a causal role in depression onset (Cruz-Pereira et al., 2020). The chronic unpredictable mild stress (CUMS) model of depressive disorder is currently the most commonly employed, effective, and reliable experimental tool to assess human psychopathology (Antoniuk et al., 2019). In a preclinical study where fluoxetine (10 mg/kg/d) was used as a positive control, intra-gastric administration of KXS water extract at the dosage of 3, 5, or 10 g/kg/day for 2 weeks remarkably ameliorated depressive-like behaviors of eight CUMS rats in sucrose preference test, forced swimming test and open field test, thereby relieving the symptoms of anhedonia, despair, and anxiety. To be more specific, the sucrose preference was markedly increased in the minimum effective dosage (5 g/kg/day) of the treated group compared with the control group (*p* < 0.05). The improvements in depressive-like behaviors seen with KXS-H (10 g/kg/d) were similar to the results seen with fluoxetine, demonstrating the efficacy of KXS on depression treatment. In parallel, this study confirmed that KXS inhibited inflammation-stimulated depression via resisting NLRP3 inflammasome activation and inducing autophagy *in vivo* and *in vitro*, as evidenced by the decrease in tumor necrosis factor (TNF-α), interleukin (IL)-6, IL-1β mRNA expression and IL-1β protein expression, as well as the accumulation in ROS content (Yu et al., 2021).

KXS was beneficial for modulating the metabolite pathway of neurotrophins (Zhu et al., 2017), inducing synaptic protein expressions (Zhu et al., 2016), and regulating pro-inflammatory cytokines in microglia (Qu et al., 2021), which might account for the concrete regulation mechanism. According to a network pharmacology investigation, 4 compounds including Asarone, Pachymic acid, Ginsenoside Rg1, and Polygalaxanthone III were identified as the key active components of KXS for treating depression (Du et al., 2022). These compounds might target 6 target genes that were closely related to four pathways, such as the serotonergic synapse pathway, neuroactive ligand-receptor interaction pathway, MAPK signaling pathway, and PI3K-Akt signaling pathway (Du et al., 2022).

A considerable number of studies have demonstrated that depressive symptoms are the highly prevalent risk factor for cardiovascular morbidity and mortality (Carney and Freedland, 2016; Bucciarelli et al., 2020; Li et al., 2020). It has been reported that comorbid depression often occurs in patients with coronary heart disease (CHD) (Khandaker et al., 2019), and the comorbidity complicates the depression treatment and worsens the cardiovascular prognosis (Carney and Freedland, 2016). TCM theory specifies that “Heart Governs the Spirit Light”. This means that a dysfunctional heart correlates strongly with mental diseases, such as insomnia, depression, and insanity (Ong et al., 2018). According to one study, KXS water extract (370 mg/kg) provided obvious protection against postmyocardial infarction depression (Hu et al., 2020). Compared to the model group, KXS treatment dramatically improved the levels of ejection fraction and fractional shortening, reduced the levels of lactate dehydrogenase (LDH) and creation kinase MB (CK-MB), increased the activities of catalase (CAT), superoxide dismutase (SOD) and glutathione peroxidase (GSH-PX), and decreased malondialdehyde (MDA) content. In addition, KXS diminished the levels of IL-6, TNF-a, and C-reactive protein (CRP) in myocardial tissue (Hu et al., 2020). Nevertheless, these experiments have obvious limitations in that only one or two doses of KXS were applied, and thus the information on the dose-dependent effect is restricted. As a classical prescription for the treatment of depression, KXS regulates prominent components of gut and brain functions (Cao et al., 2020). The anti-depression molecular mechanisms of KXS remain to be elucidated. The regulatory effects of KXS by which the microbiota-gut-brain axis affects depression are worth studying.

### 3.3 Effect on multi-infarct dementia

Multi-infarct dementia (MID), which is characterized by neuronal degeneration and multiple lesions in the cerebral gray-white matter, is the most common type of vascular dementia (VD), accompanied by obvious brain tissue pathological damage and cognitive dysfunction (Jellinger, 2013). Accumulated excitatory amino acid (EAA) and flawed energy metabolism in the brain are usually considered two major pathological hallmarks in the progression of MID (Popa-Wagner et al., 2015). Compared with the model group, pretreated with oral KXS water extract (2.12, 1.06 g/kg, 0.7 mg/kg hydergine was used as positive control) for 45 days significantly improved cognitive impairment and hippocampal neuron damage of MID rats, rescued mitochondrial functions by upregulating brain energy, ameliorating mitochondrial swelling as well as improving mitochondrial membrane potential under chronic hypoperfusion conditions, suppressed glutamate neurotoxicity through decreasing the concentration of glutamate and the level of p-NMDAR1, and noticeably activated Shh/Ptch1 signaling pathway in the brain tissue of MID rats. Intriguingly, KXS protected PC12 cells against glutamate-induced neurotoxicity via Shh/Ptch1 signaling pathway (Li et al., 2021b). Nevertheless, further in-depth clinical trials should be conducted to investigate the effectiveness of KXS as a treatment for MID.

### 3.4 The effects on cognitive functions

Cognitive dysfunction (CD) is characterized by abnormalities in reduced memory, learning, calculation, attention, comprehension, executive functioning, and psychomotor speed (Knight and Baune, 2018; Xie et al., 2020), along with abnormal mental behaviors, which interfere with patients’ daily performance and social abilities (Moussa-Pacha et al., 2020). It is well known that the Morris water maze (MWM) and Y-maze tasks are behavioral experiments designed to determine the spatial memory and learning functions (Wang et al., 2021d).

Appropriate animal models could serve as useful tools for experimental studies of drugs (Zhou et al., 2022b). Scopolamine is a pharmacologic agent that causes serious damage to cognition and functional imaging in various kinds of animals, including rats, mice, dogs, and cats (Klinkenberg and Blokland, 2010), similar to what is seen in cognitive dysfunction in humans. A substantial number of preclinical studies have employed scopolamine as a standard drug for inducing cognitive impairment models in healthy animals (Deng et al., 2019; Amirahmadi et al., 2022). Sleep deficiency or loss leads to the decline of emotion, immunity, memory, and learning (Krone et al., 2021). The rodent models of sleep deprivation show anxiety-like behaviors and cognitive impairment similar to humans (Yin et al., 2017). It is well known that cancer-related disruptions in cognitive function and memory are a common adverse effect of adjuvant chemotherapy (Speidell et al., 2018). Clinical and preclinical studies have indicated that KXS exerts a beneficial effect on CD in different animal models. In twelve scopolamine-induced CD mice, oral administration of 0.7, 1.4, and 2.8 g/kg KXS water extract (3 mg/kg/d of donepezil was used as positive control) for 2 weeks ameliorated learning and memory impairments (shortened the escape latency and increased residence time in target quadrant and the number of target crossings in MWM test, increased the percentage of alternations between the labyrinth arms in the Y-maze), decreased neuronal apoptosis and reversed neuronal degeneration (inhibited the formation of apoptotic bodies and expression of pro-apoptotic protein Bax, and upregulated the expression of anti-apoptotic protein Bcl-2), regulated cholinergic neurotransmitters (increased acetylcholine levels and the activity of choline acetyltransferase), and alleviated oxidative stress damage in the cortex and hippocampus (increased the level and activity of superoxide dismutase and glutathione peroxidase, reduced the level and activity of reactive oxygen species and malondialdehyde) (Xu et al., 2019). Treatment with oral KXS water extract (at daily doses of 500, 250, and 125 mg/kg body weight, 30 mg/kg modafinil was used as positive control) for 14 days improved learning and memory function of paradoxical sleep deprivation (PSD)-induced CD rats by decreasing the glutamic acid (GLU) and γ-aminobutyric acid (GABA) levels and modulating of the expression of some genes in the brain tissue, such as BDNF, cyclic AMP response element binding protein (CREB), and phosphorylated-CREB (p-CREB). The minimal active dose was 250 mg/kg, while the memory enhancement effect of 500 mg/kg KXS was equally effective as that of 30 mg/kg modafinil (Hu et al., 2013). Another study (0.1 mg/kg of huperzine A was used as positive control) showed that intragastric administration of 0.3 or 0.6 g/kg of KXS water extract for 2 weeks improved memory deficit induced by simulated weightlessness in ten rats mainly by inhibiting oxidative stress injury (Qiong et al., 2016). Chemotherapy-related cognitive impairment has been observed in 15% and 50% of oncology patients (Rummel et al., 2021). The therapeutic effect of KXS (at the dosage of 1 g/kg daily by gavage over 3 weeks) was studied on doxorubicin (DOX)-related cognitive impairment in 4T1 breast cancer mice. This study suggested that KXS attenuated DOX-induced cognitive impairment by regulating inflammatory responses, decreasing oxidative stress, and reversing neural degeneration. Additionally, it is worthy that KXS is beneficial to enhance quality of life and prolong survival time in DOX-treated tumor-bearing mice (Lyu et al., 2021). In summary, KXS is a promising prescription for the treatment of memory decline. These results gave inspiration for the future development of commercial agents. Nonetheless, studies investigating the clinical pharmacokinetic parameters and safety evaluation of KXS are scarce, which limits further understanding of the medicinal substance of KXS.

### 3.5 Anti-fatigue effects

Chronic fatigue syndrome (CFS) is a complex disease characterized by extreme fatigue with associated symptoms of anxiety, depression, pain, and various somatic complaints (Zhang et al., 2022b). The forced wheel running model is a popular model that has been used to simulate the chronic fatigue syndrome (Toval et al., 2021). Cao et al. evaluated the effects of KXS on CFS mice induced by forced wheel running. Briefly, 72 male Kunming mice were randomly separated into six groups (*n* = 12), including a control group, CFS group, Modafinil-treated CFS group (13 mg/kg/day), and three KXS-treated groups (175, 350 and 700 mg/kg/day). The mice in the control group were not forced to run. Correspondingly, the animals in the other five groups were induced by forced wheel running for 4 weeks. All the mice were anesthetized by intraperitoneal injections of ketamine (80 mg/kg) and xylazine (4 mg/kg). Blood was taken using a disposable syringe from the heart. The liver and muscle of mice had been weighed precisely as instructions. Levels of serum urea nitrogen (SUN), serum lactate dehydrogenase (LDH), liver glycogen (LG), muscle lactic acid (MLA), muscle glycogen (MG), and the expressions of IL-2, and IL-4 secreted by splenocytes were detected. The results showed that electric shock time was prominently reduced in the three KXS-treated groups when compared with the CFS group without drug treatment. Interestingly, the reduction in MLA, SUN, LDH and IL-4 levels and an increase in LG, MG, MLA, and IL-2 levels were demonstrated in these three KXS-treated groups than those in the CFS group without drug treatment (Cao et al., 2012). This elucidates that KXS exhibits comprehensive anti-fatigue effects. However, the specific mechanisms remain unclear, and further investigation is required to perform at animal, cellular, and molecular levels.

## 4 Pharmacokinetic studies

Pharmacokinetics (PK) investigation mainly focuses on the changes in the process of medicine absorption, distribution, metabolism, and excretion (ADME) processes *in vivo* after oral administration, and provides a helpful approach to confirm the effective ingredients and guide the clinical usage of herbal medicine (Tang et al., 2022). There are few investigations on the PK of whole KXS extracts. Currently, efficient technologies employed in the PK study of KXS are LC-MS/MS, Q-TOF/MS, HPLC-MS/MS, and UPLC-MS/MS, which are used for the determination of rat samples. The parameters are summarized as follows. In preclinical experiments, rats were given oral KXS (10 g/kg), and there were 26 prototype components (ginsenosides, polygala saponins, oligosaccharide esters, terpenoids, sucrose ester, xanthone, and other compounds) in the plasma of rats were assigned for identification using UHPLC-Q-TOF-MS. These components were derived from PR, GR, and Poria (Zhang et al., 2015). Wang et al. (2017a) obtained systematic PK data concerning the activity of KXS in the context of AD by Ultra-fast liquid chromatography-MS/MS. In normal control group, the C_max_ of POL, SB, Te, A5, A6, tenuifoliside A (TenA), GRe and GRb1 were 51.57 ± 19.10, 75.30 ± 9.10, 133.9 ± 25.3, 155.4 ± 30.8, 129.2 ± 22.9, 72.94 ± 35.17, 75.47 ± 17.2, and 269.3 ± 12.3 ng/mL, respectively. The T_max_ were 0.36 ± 0.12, 0.28 ± 0.17, 0.39 ± 0.09, 0.21 ± 0.15, 0.28 ± 0.17, 0.15 ± 0.10, 0.40 ± 0.20, 0.50 ± 0.28 h, respectively. And the area under the curve (AUC 0-∞) were 198.2 ± 52.7, 159.2 ± 58.1, 692.2 ± 322.7, 270.4 ± 69.1, 350.6 ± 103.3, 111.4 ± 44.1, 297.5 ± 42.2, and 4172 ± 902 ng h/mL, respectively. Compared with the normal group, the PK behaviors of SB, Te, and GRe in the AD model significantly illustrated a trend of continuous changes, such as higher bioavailability, better assimilation effect, slower elimination rate, delays in reaching the C_max_ and longer mean dwell time.

The basic feature of TCM is that it consists in the form of preparation and formulation by the guidance of compatibility rules, different herbs are usually orchestrated to form a multi-herbs combination. A classical prescription generally contains primary (called “Jun” or “Emperor”) and adjuvant herbs (called “Chen, Zuo, and Shi”) according to the therapeutic characteristics of TCM, and the latter function as “Assistant”, “Vassal” and “Delivery servant” which are generally worked as assistants to increase efficiency, enhance the synergistic effect or to facilitate the delivery of the emperor medicine (Wang et al., 2021a). In the TCM system, the incorporation of a “Delivery servant” into classical prescription is an important compatibility mechanism to support the transportation from the effective constituents to the target organ in treating diseases (Wei et al., 2018). ATR is assumed to act as a “delivering servant” in a multi-herb classical prescription for treatment of the central nervous disease, capable of facilitating the delivery of bioactive ingredients to the brain. KXS is a classical prescription with various PK interactions among its multi-constituents. In the case of KXS, a combination of PR, Poria, and ATR has synergistic effects on enhancement to the nootropic effects of GR. In 2010, Wang et al. investigated the PK profiles and brain distribution of ginsenosides Re, Rb1, and Rg1 after oral administration of KXS with or without ATR to rats. The result indicated that ATR in KXS increased the brain concentrations and bioavailabilities of ginsenosides Re and Rg1, but unlikely influenced the brain-to-plasma AUC ratios. ATR, the presence of the delivering servant, appeared to promote the initial absorption rate of ginsenoside in the gastrointestinal tract (Wang et al., 2010). Similar phenomena were found in the study of investigating the differences of uptaken of ginsenoside and triterpenes after oral administration of single herb and drug pairs in KXS by PK studies *in vitro* Caco-2 cell monolayers model (Zheng et al., 2018).

## 5 Clinical applications

According to TCM, depression is known as “Yu Zheng” which is manifested as a mental illness induced by insufficiency of xin-qi, stagnation of vital energy, exuberance of dampness, and phlegm in the body (Zhang and Cheng, 2019). KXS is one of the classical prescriptions for the treatment of depression. The prescription nourishes the heart, tonifies xin-qi, eliminates dampness, and tranquilizes the spirit (Yu et al., 2021). In the clinic, KXS intervention (3.2 g/d) was significantly successful in improving depressive symptoms and cognitive dysfunction in a randomized, double-blind, placebo-controlled trial involving 134 patients diagnosed with depression (Hu et al., 2021a). In addition, Hu et al. (2021b) conducted a clinical observation of 156 patients to investigate the therapeutic impact of KXS (3.2 g/d) combined with fluoxetine (20 mg/d) in the treatment of depression. Combining KXS drastically ameliorated the Hamilton Depression 17 (HAM-D17) rating scores and self-rating depression scale scores, and the serum lipoprotein B, apolipoprotein C3 and albumin levels and low-density lipoprotein cholesterol/high-density lipoprotein cholesterol ratio (Hu et al., 2021b).

## 6 Safety evaluation

Notably, rare investigations have reported the safety evaluation of KXS in preclinical and clinical practice. One report in 2011 indicated that at doses consumed in traditional medicine, KXS may be relatively safe (Mu et al., 2011). To evaluate the preclinical safety of KXS, mice received oral administration of single doses (19.67, 24.59, 30.74, 38.42, 48.03 and 60.04 g/kg, 0.8 mL/mice/time, twice/day) of the water extracts for 7 days in a study on acute toxicity, rats were given oral extracts (1, 3 and 9 g/kg, respectively) for 91 days in a study on sub-chronic toxicity. The study found that the oral median lethal dose (LD50) was 32.59 g/kg, and the mortality rate and adverse effects were clearly dose-dependent in female and male mice. In addition, the no-observed-adverse-effect level (NOAEL) of the extract was 19.67 g/kg for both sexes. No treatment-related clinical signs, mortalities, body weights, food consumption, blood chemistry, and organ parameters (brain, kidney, spleen, adrenal glands, testis, and epididymis) were observed during the sub-chronic toxicity study (Mu et al., 2011).

However, the assessment of the safety of whole classical prescription also depends on the presence of herbs with known toxicity. One of the herbs in KXS that requires monitoring is PR. PR can cause mild nausea and is regarded as a gastrointestinal side effect in routine clinical usage (Pharmacopoeia Commission of the People’s Republic of China, 2020). Long-term and excessive doses of raw PR can severely suppress gastrointestinal movement, damage the stomach and small intestine, cause intestinal wall thinning, and damage the gastric wall cell structure (Wang et al., 2018). One study showed that the use of a high dose of onjisaponin B (200 mg/kg *via* oral administration) which is derived from PR, can induce gastrointestinal congestion and swelling in mice. And the gastrointestinal irritation was decreased when PR was fried with honey (Wen et al., 2015). It is worth noting that the existing studies have indicated that ATR-derived volatile oil could noticeably depress the pulse frequency of cardiac myocytes, and slow down the heart rate at the therapeutic dose (Wu et al., 2009). In addition, α-asarone and β-asarone derived from ATR may have hepatotoxicity, genotoxicity, and carcinogenicity (Chellian et al., 2017). Although this does not indicate that KXS might lead to this toxicity, further preclinical and clinical evidence should be required to confirm the safety through long-term monitoring of the undesired effects of KXS.

The remaining herbs including GR and Poria which have been used both as food and medicine, are generally acknowledged as safe for nutrient supplements and treatment of diseases (Lu et al., 2021a; Li et al., 2022). Nevertheless, the clinical efficacy and safety of herbal medicine are also susceptible to being influenced by germplasm resources and growth conditions (Ren et al., 2020). Pesticide residues in herbal medicine are one of the most important issues affecting safety (Yue et al., 2022). More attention should be paid to the pesticide residues that may accumulate with long-term consumption of KSX.

## 7 Discussion and conclusion

Classical prescriptions are characterized by significant advantages of their high efficiency, low toxicity, minor side effects, and economic benefits (Liu et al., 2023). KXS, as an outstanding classical prescription applied for the treatment of dementia and morbid forgetfulness, has attracted substantial research interest in China and other countries in East Asia (Shan et al., 2023b). In this review, we systematically summarized the recent advances of KXS on chemical constituents, pharmacological effects, pharmacokinetics, clinical studies, and safety. Previous studies have shown that KXS is a multi-constituent, multi-target and multi-pathway classical prescription with neuroprotective, anti-AD, anti-depressant and anti-fatigue effects. It is commonly used clinically for the treatment of depression and memory decline (Qiong et al., 2016). The PK-based determination of these exposure constituents, together with metabolites after KXS administration will facilitate uncovering bioactive ingredients responsible for the therapeutic action of classical prescription (Zhang et al., 2015). All these investigations have constructed a strong scientific foundation for further research on KXS. However, the following issues still exist and remain to be further studied.

First, although many studies have demonstrated its broad therapeutic potential, the medicinal substances in the classical prescription are incompletely characterized, and the study is limited to the research on some specific and representative chemical ingredients as well as their structure-activity relationships with these bioactive ingredients. For example, the saponins from PR, the ginsenosides from GR, the phenolic acids from Poria, and the volatile oils from ATR (Li et al., 2018b). However, for some components with low contents and the bioactive compositions with high molecular weight, there are fewer studies on KXS, such as the sterols from Poria, the phenylpropanoids from ATR, as well as the polysaccharides from Poria and GR.

Second, for pharmacokinetic studies. In addition to the general analysis of ADME, numerous gut microbial play an important role in herb-taking effects (Gong et al., 2020). Yet the interaction between gut microbiota in the host and bioactive ingredients of KXS remains unclear.

Third, For quality control it is known that classical prescription is notable for its comprehensive medical effects with various complex chemical components (Li et al., 2018). Therefore, discovering and confirming the quality control markers that could reflect the overall efficacy of KXS is another important problem requiring further study.

Fourth, For pharmacological studies. Due to its unique multi-component and multi-target effect of classical prescription, the detailed molecular mechanism is absent or incomplete in most of the pharmacological studies as well as the following validation experiments. And thus in the future, the application of new technologies and research strategies should be carried out. In addition, the current pharmacology studies on quality controls including animal grouping, administration dosage, and positive drug are not strictly conducted.

In light of the question listed above, some solutions are proposed to solve these problems in the study of this classical prescription.

(I) Comprehensive chemical ingredients analysis for KXS should be further studied. More efficient and high-quality analytical techniques such as high-resolution mass spectrometry techniques, multi-dimensional chromatography, and fingerprint analysis should be applied to clarify the structure-activity relationships.
(II) KXS can replenish Qi and nourish the heart. Interestingly, the functions of the brain and gut are respectively attributed to the functions of the heart and spleen in TCM (Zhang et al., 2022a). Thus, the interaction of gut microbial and bioactive ingredients of this prescription is a promising and worthy focus.
(III) It is necessary to analyze the mechanisms of KXS and enhance the quality control markers based on the bioactive ingredients from this classical prescription. Therefore, novel methods and ideas, such as high-throughput screening in conjunction with clinical outcomes, experimental validations after the network pharmacology definition, and metabolomics analysis should be adapted to further research. It helps to analyze the complex mechanism of action of KXS in various diseases and expand its indications.
(IV) It is worth noting that the kinds of major active components and their contents vary based on the habitat of medicinal plants. The quality of medicinal plants plays an essential role in ensuring the clinical efficacy and safety of classical prescription. Thus, it is necessary to apply genuine Chinese medicine and/or the medicinal plant cultivated in the main regions to control the quality of KXS. Moreover, the development of robust quality control strategies should be applied to the overall evaluation of KXS quality.

Taken together, the present review has concluded with a summary and insight into the basic information about KXS, which might provide a scientific basis for the related research of KXS. Additionally, this paper contains a brief introduction about the potential value and some new research directions on KXS, thereby providing new research ideas for better exploration and exploitation in the future.

## Figures and Tables

**Table:** 

**KXS**	Kaixinsan
**GRe**	Ginsenoside Re
**GRb1**	Ginsenoside Rb1
**POL**	Polygalaxanthone III
**TCM**	Traditional Chinese medicine
**LC**	Liquid chromatography
**MS**	Mass spectrometry
**QTOF-MS/MS**	Quadrupole time-of-flight tandem mass spectrometry
**GC**	Gas chromatography
**UPLC**	Ultra-performance liquid chromatography
**POL**	Polygalaxanthone III
**Abeta**	Amyloid beta peptide
**AChE**	Acetylcholinesterase
**IL**	Interleukin
**BDNF**	Brain derived neurotrophic factor
**HPA**	Hypothalamic-pituitary-adrenal
**CUMS**	Chronic unpredictable mild stress
**CHD**	Coronary heart disease
**MI**	Myocardial ischemia
**LDH**	Lactate dehydrogenase
**CK**	Creation kinase
**CAT**	Catalase
**SOD**	Superoxide dismutase
**GSH-PX**	Glutathione peroxidase
**MDA**	Malondialdehyde
**AD**	Alzheimer’s disease
**CRP**	C-reactive protein
**MID**	Multi-infarct dementia
**VD**	Vascular dementia
**CD**	Cognitive dysfunction
**MWM**	Morris water maze
**PSD**	Paradoxical sleep deprivation
**GLU**	Glutamic acid
**GABA**	γ-aminobutyric acid
**CREB**	Cyclic AMP response element binding protein
**DOX**	Doxorubicin
**CFS**	Chronic fatigue syndrome
**SUN**	Serum urea nitrogen
**LG**	Liver glycogen
**MLA**	Muscle lactic acid
**MG**	Muscle glycogen
**PK**	Pharmacokinetics
**ADME**	Absorption, distribution, metabolism and excretion
**Ach**	Acetylcholine
**DA**	Dopamine
**5-HT**	5-hydroxytryptamine
**5-HIAA**	5-hydroxyindoleacetic acid
**PI3K**	Phosphoinositide 3-kinase
**Akt**	Protein kinase B
**ROS**	Reactive Oxygen Species
**TNF-α**	Tumor necrosis factor-alpha
**AA**	Arachidonic acid
**Bax**	BCL2-associated X protein
**Bcl-2**	B cell lymphoma 2
**BHT**	Butylated hydroxytoluene
**AMPK**	AMP-activated protein kinase
**SAMP8**	Senescence-accelerated mouse prone 8
**NLRP3**	Nucleotide-binding oligomerization domain-like receptor family, pyrin domain containing 3
**MMP**	Mitochondrial membrane potential
**EC**	Energy charge
**NMDAR1**	N-methyl-D-aspartate receptor 1
**ChAT**	Choline acetyltransferase
**SYN**	Synaptophysin
**p-CREB**	Phosphorylated-CREB
**8-OHdG**	8-hydroxy-2′-deoxyguanosine
**3-NT**	3-nitrotyrosine
**CUMS**	Chronic unpredictable mild stress
**m-TOR**	Mammalian target of rapamycin
**BLA**	Lactic acid
**CK-MB**	Creation kinase MB
**NE**	Epinephrine
**NGF**	Nerve growth factor
